# SWOT Analysis and Preliminary Study on Prevention and Control Management of Temporary Integrated Isolation Ward During COVID-19 Outbreak

**DOI:** 10.3389/fpubh.2021.558565

**Published:** 2021-03-15

**Authors:** Ye Zhou, Lixiang Bai, Hao Guo, Shaowei Guo, Xiaowen Han, Ning J. Yue, Qingxia Li

**Affiliations:** ^1^The Fourth Department of Oncology, Hebei General Hospital, Shijiazhuang, China; ^2^The Department of Respiratory, Hebei General Hospital, Shijiazhuang, China; ^3^Department of Radiation Oncology, Rutgers Robert Wood Johnson Medical School, New Brunswick, NJ, United States

**Keywords:** COVID-19, SWOT analysis, temporary integrated isolation ward, prevention and control management, hospital management

## Abstract

**Background:** The world faced crises of prevention and control and shortage of medical resources during the COVID-19 (Corona Virus Disease 2019) outbreak. The establishment of temporary integrated isolation wards in hospitals, which is universal and representative in China, is one of the most-effective strategies in solving these problems according to China's experiences.

**Aim:** To conduct a preliminary study on the establishment of a temporary integrated isolation ward during the outbreak of COVID-19 and to evaluate related impact.

**Methods:** SWOT analysis was used to analyze the advantages, disadvantages, opportunities, and risks in the establishment of the temporary integrated isolation ward, and corresponding corrective measures were made according to the analysis results.

**Findings:** The ward has formulated more than 10 related work procedures and prevention and control measures. A total of 93 patients with 18 critically ill patients were admitted for treatment and isolation. They were all evaluated based on established procedures and protocols. Twenty-four supplementary nucleic acid tests were ordered and conducted. One new patient with COVID-19 was confirmed and was successfully transferred to the designated COVID-19 infectious control hospital. There were no missed diagnosis or misdiagnosis, no cross-infection of patients, no cluster outbreak, and no infection of medical workers during the entire process.

**Conclusion:** SWOT analysis is helpful in guiding the establishment of a temporary integrated isolation ward and the formulation of prevention and control measures in Hebei General Hospital during the COVID-19 outbreak. It provides the guidance and reference of significance for the establishment of similar types of wards in the future.

## Introduction

COVID-19 is an acute respiratory b infectious disease mainly caused by a novel coronavirus (SARS-cov-2) and is characterized mostly by pulmonary inflammatory lesions (1, 2). According to the data released by the Chinese national health commission, there were 15,152 confirmed cases and 2,807 suspected cases by February 12, 2020 in China (3), hitting a new high for the COVID-19 infected patients in China. Hebei General Hospital is a Level 1 first-class comprehensive hospital directly directed by the Department of Health of Hebei Province of China and undertakes important epidemic prevention and control work in Hebei Province. To respond to the COVID-19 outbreak and the potential impact to the care of febrile patients and health-care workers, on February 13, 2020, Hebei General Hospital decided to transform the original Fourth Department of Oncology, which is a comprehensive department managing cancer patients with radiotherapy and chemotherapy, into a temporary integrated isolation ward. The focus of the newly established department is to manage various patients under observation with fever and conduct the screening and isolation of suspected COVID-19 infected patients, as well as other related work. Because the hospital had no previous experiences of temporary integrated isolation wards, it is particularly important to explore the establishment of plans for temporary integrated isolation wards and subsequent management issues in order to complete epidemic prevention work with high quality (4).

SWOT analysis refers to the assessment and evaluation of various strengths (S), weaknesses (W), opportunities (O), threats (T), and other factors that influence a specific topic. It comprehensively, systematically, and accurately describes the scenario in which the topic is located. This method can be used to identify favorable and unfavorable factors and conditions, solve current problems in a targeted manner, recognize the challenges and obstacles faced, and formulate strategic plans to guide scientific decisions. It can be useful in a scientific field and in the medical management field (4–10). This methodology was adopted in the establishment of the temporary integrated isolation wards in our hospital. This paper presents the SWOT analysis in our hospital that analyzed the current situation of establishing temporary integrated isolation wards and formulated corresponding rectification and prevention and control measures according to the results.

## Methods and Analysis

### Methods

The qualitative study was conducted according to the Consolidated Criteria for Reporting Qualitative Studies guidelines (11). The semi-structured focus groups were conducted by the Epidemic Prevention and Control Team of Hebei General Hospital from February 1, 2020 to February 12, 2020. It aimed to explore respondents' experiences and insights on the establishment of the temporary integrated isolation ward in our hospital. Twelve respondents of this study were selected purposively based on their profession in the hospital, inclusive of the staff of administrative departments of the hospital (Group A), members of the infection prevention and control group (Group B), staff of the Fourth Department of Oncology (Group C), and other clinical technical departments (Group D). Based on the guideline, these members were purposely selected as the sampling in this study because of their important positions in their respective professional fields.

Some questions and prompts were applied to data collection by the Epidemic Prevention and Control Team during their interview to encourage respondents to openly convey their viewpoints, such as What is the significance of converting the Fourth Department of Oncology into temporary isolation wards? How do you avoid the risk of cross-infection? How does the mental state of medical personnel adjust? How is the patient managed after entering the isolation ward? What support should the hospital supply? What unknown risks will the hospital and temporary isolation wards face and how to avoid them? The semi-structured focus groups were conducted by face-to-face interviews and lasted about 90 min every time. Interviews were repeated twice.

The members' opinions were collected during the interviews. A coding procedure was done by Dr. Han, Dr. Li, and a member of the Epidemic Prevention and Control Team. The same code was given to the similar opinions. They achieved an agreement on a set of codes that would be used for the analysis through a discussion among them. Then, the Epidemic Prevention and Control Team discussed the codes and generated the themes for each code according to the interrelation of similar opinions and the subcodes' counts of each code. The themes were compiled according to the strategic planning analysis of strength, weakness, opportunity, and threats (SWOT). Rank the importance of codes by counts. Recode from 1 in order of importance. The themes were entered separately in each factor of SWOT according to the new encoding. The data was summarized using a framework matrix. Strategic planning was specified according to the relationship between the data in the framework (11, 12) (Table 1).

**Table 1:** Stakeholders' view of the establishment of the temporary integrated isolation wards in our hospital: SWOT Analysis.

**Factor**	**Content**			
Strengths	1. The hospital has the capacity and support to establish isolation wards	2. The location of the department is independent	3. Medical staff have been trained in professional skills	
Weakness	1. The ward facilities do not meet infection control standards	2. It is difficult for The Fourth Department of Oncology to develop work procedures for isolation wards	3. The number of medical staff is insufficient and the professionals are not suitable	4. Patients and families are under great psychological pressure
Opportunity	1. The establishment of temporary isolation wards contributed to the epidemic prevention and control in Hebei province and the whole country	2. The comprehensive strength of The fourth Department of Oncology will be improved after they undergo rectification	3. Ensure the safety of febrile patients and relieve the pressure of other departments	4. Improve information construction of the hospital
Threat	1.Inadequate conditions in isolation wards pose a risk of cross-infection	2. The exploratory isolation ward has less experience but more difficulties	3. Complex conditions in admitted Patients can lead to medical care errors	

*S, strengths; W, weaknesses; O, opportunities; T, threats*.

### SWOT Analysis

#### Strength Analysis

(1) Internal advantages of the hospital: Hebei General Hospital has a sound hospital infection management system and related streamlined policies and procedures, strong routine emphasis in the importance of epidemic prevention, and strong leadership supports and leads to the establishment and operation of temporary integrated isolation wards. (2) Internal advantages of the original department: the department is located near the west gate of the hospital, which is an independent three-story building. It is far from the main ward area and close to the open streets. It has good ventilation system, and its geographical advantages are conducive to the prevention and control of cluster-infection and the realization of secondary isolation. (3) Advantages of professional skill and knowledge training: the whole hospital has excellent communication and training networks, such as hospital internal information network, “good doctor” network, and WeChat group; daily morning session has been set up to conduct knowledge and skills training and assessment of COVID-19 cases. Before receiving the task of integration, our department staff members acquired sufficient relevant knowledge and passed the training examination proctored by the hospital and Department of Health.

#### Weakness Analysis

In terms of ward facilities, the building in our section was built many years ago and is not in accordance with the current requirements of the professional isolation ward. There was no access control system, no perfect “three areas and two passages” structure, and no adequate air conditioning and professional ventilation system, so there was risk of cross-infection and cluster-infection.

Two aspects of weakness in workflows include: (1) The department was originally a general oncology department, but the internal workflow had to be changed according to the isolation ward standards after rectification. Due to lack of relevant experiences, the ability to formulate and implement the isolation ward work process was relatively weak. (2) In order to minimize the contacts between patients and their families with the outside world, medical staff would take the place of or lead the work of handling the hospitalization procedures, patients going out for inspection, taking medicine, sending samples, sending meals, registering and paying fees, etc., for patients. These extra loads of work on the health-care workers of the department are huge and complex and require careful planning.

Two aspects of weakness in human resources are: (1) The department was originally staffed with 22 doctors and nurses, all of whom were engaged and specialized in cancer management and care. They were not specially trained for taking care of COVID-19-infected patients. And after the rectification, the department needed to be divided into three layers of support, with a sharp increase in workload, high practicing pressure, and insufficient manpower. (2) Most of the department staff members are young medical care professionals, with relatively few experiences in handling public health emergencies, disaster rescue, etc., and a weaker ability in risk management and first aid than the dedicated infectious control and ER health-care workers.

Patients and companions: most of the patients admitted and treated in the department have complicated and severe diseases. The immune function of the patients is normally lower than that of ordinary patients, and their ability of self-protection and isolation protection is lower, leading to possible increased risk of cross-infection, and the psychological pressure on the patients and their companions is also likely greater.

#### Opportunity Analysis

(1) For the prevention and control of COVID-19, it was the first attempt of such kind in the history of the entire hospital and the department. The established temporary integrated isolation ward will contribute to the prevention and control of the epidemic in Hebei Province and even the whole country. It will provide valuable experiences for the establishment of similar departments in the future. (2) The department rectification is fully supported by the hospital. Its successful completion will significantly improve the department management ability, the ability to deal with major emergencies, and the professional ability of medical staff, laying a solid foundation for the rapid development of the department in the future. (3) The establishment of a temporary integrated isolation ward will help the diversion of patients with fever and greatly reduce the pressure of infection and emergency departments. It can better guarantee the personal safety of patients with fever and reduce the risk of infection of other departments in the hospital. (4) During the epidemic outbreak period, most offline continuing education programs were canceled, and various forms of online education activities have been developed for the completion of the project. This provides great opportunities for the construction of online network information-based telemedicine in our hospital.

### Risk/Threat Analysis

(1) The department will be a post-rectification ward, and the prevention and control conditions of the ward environment and equipment are still relatively poor compared to those of the professional department of infection diseases, and there are hidden risks such as potentially increased nosocomial infection and spreading infection. (2) After the rectification of the department, the department mainly accepts and treats the febrile patients for observation and the critically ill patients. Patients' conditions and illnesses vary and can be very complicated. The department will be faced with many challenges, such as the exploratory ward reformation, the development of new workflows, isolation and protection, professional skills training, and other peripheral works. The fact that the original Fourth Department of Oncology lacked the professional skills, experiences, and human resources required for the special isolation ward may lead to some medical care errors, redundant work, and psychological pressure on the medical staff (Table 2).

**Table 2:** SWOT matrix (2 × 2) of the temporary integrated isolation ward of in Hebei General Hospital.

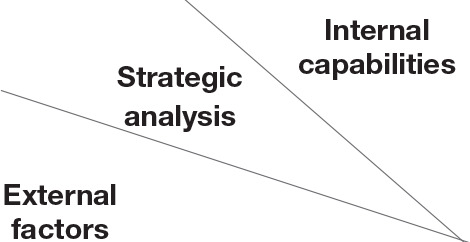	**Strength**	**Weakness**
	1) The hospital has the capacity and support to establish isolation wards 2) The location of the department is independent 3) Medical staff have been trained in professional skills	1) The ward facilities do not meet infection control standards 2) It is difficult for The fourth Department of Oncology to develop work procedures for isolation wards 3) The number of medical staff is insufficient and the professionals are not suitable 4) Patients and families are under great psychological pressure
**Strategic analysis**		
**External factors**		
Opportunity	SO strategies	WO strategies
1) The establishment of temporary isolation wards contributed to the epidemic prevention and control in Hebei province and the whole country. 2) The rectification about the ward can improve the “soft power” of the department. 3) Ensure the safety of febrile patients and relieve the pressure of other departments. 4) Improve information construction of the hospital.	1) Make good use of the advantages of hospital policies to carry out the reform of ward areas. 2) Seize opportunities to carry out online and offline learning.	1) Apply for the assistance of relevant departments in the hospital. 2) Draw lessons from the experience of other hospitals. 3) Summarize the appropriate rectification methods. 4) Formulate the process system.
Risk/threat	ST strategies	WT strategies
1) Inadequate conditions in isolation wards pose a risk of cross-infection. 2) The exploratory isolation ward has less experience but more difficulties. 3) Complex conditions in admitted patients can lead to medical care errors.	1) Apply for more human resources and equipment. 2) Improve the professional prevention and control skills of medical staff, strengthen the psychological construction, and monitor the physical condition.	1) Strictly implement the new system and workflows. 2) Strengthen the prevention and control of hospital disinfection and natural ventilation of the ward to make up for the lack of conditions. 3) Strengthen the management of patients and family in all aspects.

## The Prevention and Control Strategies of the Temporary Integrated Isolation Ward

According to the strategies of SWOT analysis about the temporary integrated isolation ward of in Hebei General Hospital, some specific measures are as follows.

### Ward Reformation and Transformation, Strict Zoning, and Reasonable Layout

The original ward areas were divided according to the rule of “three areas, two passages,” that is, clean area, semi-polluted area, polluted area (three areas), and the medical staff channel, the patient channel (two passages). The south area of the first floor is the clean area with a duty room. The south area of the second and third floors are the semi-polluted areas, including the doctor's office, nursing station, treatment room, and warehouse. The north area of the second and third floors are the polluted areas. Clearly visible signs and marks are placed on the doors and walls of each of the areas for their roles and functions. The cleaning and pollution routes of people flow and logistics, which are mutually exclusive (13), are strictly regulated. There are 11 single-room isolation wards in the entire ward area, each of which is equipped with, oxygen, suction, and other bedside treatment facilities, as well as calling and intercom systems. Adequate space is allocated at the bedside for bedside X-ray machines, breathing machines, etc. All the wards are equipped with private toilets, showers, and handwashing facilities, as well as electric heating and ultraviolet disinfection lamp. Every effort is made to ensure that the wards, medical offices, and duty rooms are well-ventilated. The department is well-stuffed with protective clothing, goggles, respirator, latex gloves, alcohol, chlorine-containing disinfectant, and other disinfection and protection materials.

### Workflows and Management Protocols

According to the prevention and control strategies of COVID-19 and the specific situation of the department, specific workflows and protocols are established for the temporary integrated isolation ward. The protocols and workflows include “Patient Admission Workflow,” “Procedures of Receiving and Observing Patients with Fever,” “Notes for Medical Personnel,” “Precautions for Admission and Treatment of Patients,” “Graded Protection Requirements for Medical Personnel,” “Procedures of Peripheral Works,” “Detailed Division of Duties of Doctors on Duty,” “Disinfection Measures of COVID-19,” “Observed Patient Notification,” “Flow Chart of Entrance Personnel Check,” etc. All medical staff members, patients, and other supporting personnel were organized to study together and strictly supervised (14, 15).

Strengthen medical personnel support and human management. The hospital has reassigned 19 medical doctors and nurses to support our department, including the experts from respiratory department, department of cardiology, emergency department, and department of surgery. After the integration, there were 41 medical staff members in the department, including 18 medical doctors with eight from the oncology department, four from the respiratory department, four from the emergency department, one each from departments of cardiology and nephrology, and 23 nurses. Based on the staffing level, our department has formulated the detailed rules of division of workload and responsibilities and implemented a “group shift” scheduling system, with each shift working for 4 days, so as to ensure that each shift group includes one respiratory physician, one physician in the isolation ward, one physician in the cleaning ward, two nurses in the isolation ward, and two nurses in the semi-contaminated ward. The doctors in each shift group have worked seamlessly according to the division of workload and responsibilities. Every effort is made to ensure that there is no vacant post in each shift or no discontinuity of clinical coverage, to optimize the shift positions to avoid unnecessary congestion and to minimize the likelihood of cross-infection and to avoid work related fatigue. At the same time, efforts are made to the staff idle time to conduct emergency isolation and protection training, COVID-19-related knowledge teaching, and clinical diagnosis and treatment skills training for all staff members in the department (16).

Strengthen patient management: (1) Check the potential COVID-19 infection indications at patient admission. For patients with fever and respiratory symptoms who have had negative nucleic acid test for the first two times, they will be quarantined to the isolation room, waiting for further nucleic acid tests and lung CT tests. An investigation will be conducted for the causes of the symptoms. Consultation will be made with the members of the hospital's infectious disease prevention and control expert group if necessary. For critically ill patients requiring emergency admission, a nucleic acid and pulmonary CT examination shall be performed immediately. Patients with fever and pulmonary lesions shall be admitted to the isolation ward, and the admission can be made according to the corresponding prevention and control protocols. (2) The wards are divided into three different levels, and patients are “managed by division” according to high, medium, and low risk. Efforts are made so that one room accommodates one patient. The patients suspected of contracting COVID-19 and critically ill patients are mainly monitored (14, 15, 17). (3) Follow-up of patients discharged from the hospital is made. It is recommended that patients with fever continue to be self-quarantined at home after discharge. In the first 3 days after discharge, the on-duty doctor calls the patient for the body temperature and records the temperature in patient chart. If the patients' temperature was normal, it would be recorded every other day during the next 14 days. (4) Carefully examine and check the epidemiological exposure history of patients with COVID-19. When a patient is suspected or confirmed to be infected with COVID-19, a report is immediately filed to the relevant departments of the medical institution, and if necessary, arrangement is proactively made to transfer the patient to a designated COVID-19 control and management hospital. (5) Provide the psychological consultation and comfort to patients and their families to minimize related burdens during treatment.

Simplify peripheral workflows. Apply to the hospital to allow the department to directly process patient registration, prescription, billing, and other works in order to reduce the contacts of the medical staff, patients, and their families with the environment outside the ward and the department, thus the risk of infection. At the same time, medical workers are encouraged to work from home if possible.

### Strengthen Disinfection, Isolation, and Protection to Make Up for the Lack of Certain Needed Conditions in the Ward

(1) Disinfection of the ward shall be carried out strictly in accordance with Disinfection Measures of COVID-19 (18). In addition, the department has strengthened the prevention and control of contact and air transmission after the rectification of the environment and promptly disinfected the surfaces of objects, prescriptions, and other potential polluted objects in the department. Ensure that all staff members wear the isolation clothing correctly, enforce hand hygiene, and ensure that disinfection supplies are safe and effective. Procedures are set up to register the usage of disinfection drugs and disinfection consumables, report the information to the hospital, and apply for the personal protective equipment in a timely fashion (18, 19). When doctors are on duty, they should strictly follow personal protection in accordance with Graded Protection Requirements for Medical Personnel (20). (2) Register and record the body temperature of patients, staff members, and visitors at the entrance of hospital and department. The epidemiological investigation is conducted to the medical staff. A related questionnaire is distributed to and completed by the medical staff. To enhance the awareness, the relevant informed notice and fliers on the knowledge of prevention and control of COVID-19 are posted on the ward corridor, publicity board, and social media.

### Pay Attention to the Physical and Mental Health of All Staff

The hospital administrative team regularly visits the department staff, provides daily medical supplies, and ensures that nutritious diet is readily available. A special psychological consultation clinic is opened, and free psychological consultation is provided to medical staff. Based on the nature of job responsibilities, the daily temperature and respiratory symptoms of medical personnel are monitored and registered. When necessary, free CT and blood monitoring examinations are provided for medical personnel to ensure that medical personnel can provide medical services to patients in a healthy body and mind (21, 22).

## Preliminary Results of Prevention and Control Strategies

With the support of the hospital, the comprehensive ward of The Fourth Department of Oncology, namely the temporary integrated isolation ward, has been transformed and put into use, with reasonable and sufficient personnel professional structure and supporting and functioning capacity. The supply of protective substances and equipment is abundant. It can not only ensure the medical needs of febrile patients with different diseases but also ensure the smooth development of the prevention and control workflows of COVID-19 patient management and care. Since the temporary integrated isolation ward was set up on February 13, 2020, more than 10 related work procedures and prevention and control strategies/protocols have been preliminarily formulated and developed. A total of 93 patients with 18 cases of critically ill patients were admitted for treatment and were isolated if needed based on their symptoms. Twenty-four additional nucleic acid detection tests were ordered and completed. One patient was confirmed to be COVID-19 positive. There were no missed diagnosis and misdiagnosis, no cross infection in the ward patients, no mass outbreak, and no infection among medical workers.

The department has conducted and participated in online and offline COVID-19 prevention and control knowledge training, training and assessment of hospital and department rules and regulations, and patient and family education training and assessment. Forty such trainings and assessments were conducted, with a training participation rate and assessment passing rate of 100%. A high level of morale and spirit was observed and maintained among the medical staff.

## Conclusion

In this study, SWOT analysis is presented on the establishment of a temporary integrated isolation ward in Hebei General Hospital, and corresponding measures and protocols based on the analysis results are also presented for its operation during the COVID-19 outbreak. The study may be useful in providing guidance and reference for the similar types of wards in other hospitals and regions. At present, the COVID-19 pandemic is not over and a lot still needs to be learned. The analysis and strategies presented in this study could serve as a stepping-stone for more comprehensive management measures of pandemic infectious disease control and patient care.

## Data Availability Statement

All datasets presented in this study are included in the article/supplementary material.

## Author Contributions

YZ: methodology, formal analysis, and drafting the article. LB and HG: analysis and interpretation of data. SG: acquisition of data. XH: supervision and project administration. NY: revising it critically for important intellectual content. QL: the conception, design of the study, and final approval of the version to be submitted. All authors contributed to the article and approved the submitted version.

## Conflict of Interest

The authors declare that the research was conducted in the absence of any commercial or financial relationships that could be construed as a potential conflict of interest.
